# Predicting targeted drug combinations based on Pareto optimal patterns of coexpression network connectivity

**DOI:** 10.1186/gm550

**Published:** 2014-04-30

**Authors:** Nadia M Penrod, Casey S Greene, Jason H Moore

**Affiliations:** 1 Department of Pharmacology and Toxicology, Geisel School of Medicine at Dartmouth College, HB7937 One Medical Center Dr, Lebanon NH 03766, USA; 2 Department of Genetics, Geisel School of Medicine at Dartmouth College, HB7937 One Medical Center Dr, Lebanon NH 03766, USA; 3 Institute for Quantitative Biomedical Sciences, Geisel School of Medicine at Dartmouth College, HB7937 One Medical Center Dr, Lebanon NH 03766, USA

## Abstract

**Background:**

Molecularly targeted drugs promise a safer and more effective treatment modality than conventional chemotherapy for cancer patients. However, tumors are dynamic systems that readily adapt to these agents activating alternative survival pathways as they evolve resistant phenotypes. Combination therapies can overcome resistance but finding the optimal combinations efficiently presents a formidable challenge. Here we introduce a new paradigm for the design of combination therapy treatment strategies that exploits the tumor adaptive process to identify context-dependent essential genes as druggable targets.

**Methods:**

We have developed a framework to mine high-throughput transcriptomic data, based on differential coexpression and Pareto optimization, to investigate drug-induced tumor adaptation. We use this approach to identify tumor-essential genes as druggable candidates. We apply our method to a set of ER^+^ breast tumor samples, collected before (*n* = 58) and after (*n* = 60) neoadjuvant treatment with the aromatase inhibitor letrozole, to prioritize genes as targets for combination therapy with letrozole treatment. We validate letrozole-induced tumor adaptation through coexpression and pathway analyses in an independent data set (*n* = 18).

**Results:**

We find pervasive differential coexpression between the untreated and letrozole-treated tumor samples as evidence of letrozole-induced tumor adaptation. Based on patterns of coexpression, we identify ten genes as potential candidates for combination therapy with letrozole including EPCAM, a letrozole-induced essential gene and a target to which drugs have already been developed as cancer therapeutics. Through replication, we validate six letrozole-induced coexpression relationships and confirm the epithelial-to-mesenchymal transition as a process that is upregulated in the residual tumor samples following letrozole treatment.

**Conclusions:**

To derive the greatest benefit from molecularly targeted drugs it is critical to design combination treatment strategies rationally. Incorporating knowledge of the tumor adaptation process into the design provides an opportunity to match targeted drugs to the evolving tumor phenotype and surmount resistance.

## Background

A great deal of effort has been directed toward the identification of molecular targets that drive oncogenesis and the development of novel therapeutics that interact with these targets [1-6]. However, tumor cells have a remarkable ability to adapt to such treatments through functional redundancies and activation of compensatory signaling pathways that enable them to tolerate the presence of targeted drugs. Thus, despite making important contributions to the treatment of cancer, the success of targeted therapies has been limited by resistance.

The predominant strategy for overcoming resistance is to combine drugs that act through ancillary mechanisms to block the functional redundancies and compensatory signaling pathways that serve as escape routes for cell survival. This strategy is supported by studies showing that complex networks, including the networks of molecular interactions that underlie biological function, are vulnerable to coordinated attacks at multiple targets [7,8], and by functional genomics screens with RNA-mediated interference showing that cells can be increasingly sensitized to a molecularly targeted drug by inhibiting a second complementary target concurrently [9]. While this strategy is intuitive and may appear straightforward, selecting the best combination of targets to maximize tumor cell death while minimizing collateral damage and toxicity presents a tremendous challenge. Furthermore, it does not take into account the evolving tumor phenotype that emerges through the adaptation process in response to drug perturbation.

To address this challenge we have developed a framework to identify tumor-essential genes as potential drug targets by mining high-throughput transcriptomic data based on coexpression patterns where coexpression serves as a proxy for coregulation or participation in the same biological processes [10,11]. We apply this method to tumor samples taken from breast cancer patients undergoing preoperative letrozole treatment. This allows us to identify essential genes in the primary and residual tumors capturing changes in essentiality as the tumors adapt to the drug.

Letrozole is a non-steroidal aromatase inhibitor that binds competitively and reversibly to the aromatase enzyme and, in effect, inhibits the production of estrogen by blocking the conversion of androgens into estrogens. Estrogen regulates cell growth and differentiation influencing the development and progression of breast cancer by binding to and activating estrogen receptors (ERs). ERs participate in cell signaling and regulate gene expression through the activation or repression of gene transcription [12].

Letrozole is used neoadjuvantly to reduce the volume of large operable, locally advanced, and inoperable ER^+^ breast cancers in postmenopausal patients [13,14]. Efforts have been made to enhance the effects of letrozole by combining it with other drugs to reduce further tumor burden in responders and to develop effective treatment strategies for nonresponders [15-17]. To date, these combinations have led to only modest increases in clinical response. For example, combining letrozole with the mTOR inhibitor everolimus increases response rates, determined by clinical palpitation, at a moderately statistically significant level (*P* = 0.062) relative to letrozole treatment alone [15]. This indicates that the effects of letrozole can be enhanced by combining it with other molecularly targeted drugs, but it also suggests that there is room for improvement in choosing the most effective combinations for letrozole in this setting.

Here we assess patterns of differential coexpression among patient tumors sampled before and after letrozole treatment. Based on these coexpression patterns we identified tumor-essential genes and letrozole-induced tumor-essential genes as potential candidates for combination therapy with neoadjuvant letrozole treatment. We show that coexpression is a suitable measure of tumor adaption to drug perturbation by validating letrozole-induced coexpression relationships in an independent data set.

## Methods

### Data description

The initial analysis was performed with transcriptomic data generated from core biopsies of ER^+^ breast tumors at diagnosis (*n* = 58) and again following a 90-day course of neoadjuvant treatment with the drug letrozole (*n* = 60) [18,19]. Inclusion criteria required the samples to contain at least 20% malignant tissue. RNA was extracted, amplified, and hybridized to Affymetrix HG-U133A GeneChip arrays. The data are publicly available through the Gene Expression Omnibus (GEO) database [GEO:GSE20181].

An independent data set was used for replication. The replication data are also transcriptomic profiles generated from core biopsies of ER^+^ breast tumors at diagnosis (*n* = 18) and again following a 90-day course of neoadjuvant treatment with the drug letrozole (*n* = 18) [20]. Inclusion criteria required the samples to contain at least 50% malignant tissue. RNA was extracted, amplified, and hybridized to Affymetrix HG-U133 Plus 2.0 GeneChip arrays. The data are publicly available through GEO [GEO:GSE10281].

### Data processing

We downloaded and processed the raw probe intensity (CEL) files for each data set independently. We used a custom chip definition file (CDF) to ensure we were using the most recent probe annotations and to filter the Affymetrix probe sets to include only those probes that uniquely map to genes [21]. Data were background corrected, normalized, and summarized using the robust multi-array average algorithm [22] as implemented in the R statistical language [23].

### Differential expression analysis

Differential expression analysis between untreated and letrozole-treated tumor samples was conducted using the linear models for microarray data (limma) method [24] implemented in the limma package in R. We chose this method based on its robust performance across a variety of sample sizes and noise levels [25]. To correct for multiple hypothesis testing, genes at a false discovery rate (FDR) below 5% were considered differentially expressed at a statistically significant level. We performed coexpression analysis on the set of differentially expressed genes.

### Differential coexpression analysis

Using the subset of genes found to be differentially expressed by letrozole treatment, we generated two sets of coexpressed gene pairs, those that occur in the untreated tumor samples and those that occur in the letrozole-treated tumor samples. To identify coexpressed gene pairs, we calculated the first-order Spearman’s partial correlation coefficients and associated *P* values [26] between the expression levels for each pairwise combination of genes. Spearman’s correlation allows us to identify both linear and non-linear coexpression relationships and has been shown to outperform the more commonly applied Pearson’s correlation coefficient at identifying coexpression relationships among genes within the same pathways and among functionally related transcription factors [27].

Gene pairs with a coexpression *P* value that met an FDR-based significance threshold of *α* = 0.01 were retained. This significance threshold was chosen based on simulations carried out by de la Fuente *et al*. [26]. To validate this threshold for selecting coexpressed gene pairs in our data, we used permutation testing to model the null hypothesis that there are no coexpression relationships among genes in these data sets (Additional file 1). Permutation tests were designed to randomize the expression values for each gene, across samples, within each time point. Following randomization, we calculated coexpression as described above and counted the number of partial correlation coefficients that met our significance threshold. This process was repeated 1,000 times to generate a null distribution. The observed numbers of significant coexpression relationships, for untreated and treated tumors in both data sets, fall to the right of the upper bound in the matched null distribution (*P* < 0.001) (Additional file 1) allowing us to reject the null hypothesis by showing that more gene pairs were coexpressed than would be expected by random chance when a significance threshold of *α* = 0.01 is applied. The complete results of the coexpression analysis are presented in Additional files 2, 3, 4, 5, 6 and 7.

### Annotating coexpressed gene pairs

We annotated each gene to the Gene Ontology (GO) biological process [28], Kyoto encyclopedia of genes and genomes (KEGG) [29], and Reactome [30] databases through Bioconductor. We found common processes and pathways by intersecting the annotations for each pair of genes.

We also evaluated each gene pair for functional relationships based on empirical data with networks from the Integrated Multi-species Prediction (IMP) web server [31]. These gene networks were generated as described in Park *et al*. [32] and integrate data sources that include wet biochemical evidence including the IntAct, MINT, MIPS, and BioGRID databases. In these gene networks, edges represent the posterior probability of a functional relationship between two genes. Therefore, each edge is interpretable as the posterior probability, given a large compendium of empirical data collected from human-derived samples, that two genes work together to carry out a specific biological process. We overlaid our coexpressed gene pairs onto these networks to determine the likelihood that a functional relationship exists between the pairs of genes we identify.

For novel gene pairs that replicate, we used IMP to predict functional relationships directly and to identify bridging genes that connect coexpressed gene pairs. For this purpose we considered edges above a probability threshold of 0.70. This cutoff is stringent: only 0.042% of edges in the network (141,214 / 333,452,400) have sufficient evidence to place them above this threshold. Functional descriptions of the genes in the results were taken from GeneCards [33].

### Pareto identification of tumor-essential genes

**Figure 1:**
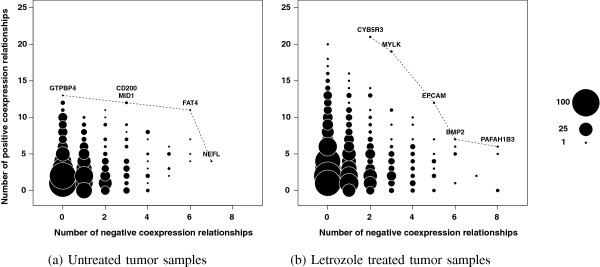
**Gene-wise patterns of connectivity reveal essential genes as potential drug targets for combination treatment with letrozole.** Using Pareto optimization we identified the set of genes that fall along the Pareto front denoted by the dashed lines in the **(a)** untreated and **(b)** letrozole-treated tumor samples. These are the genes that have the optimal balance of positive and negative connections, a property that has been associated with essentiality.

Studies in model organisms demonstrate that essential genes tend to have a combination of many positive and many negative genetic interactions [34]. Based on these findings we used coexpression as a proxy for coregulation and we identified essential genes as those that have many positively and many negatively coexpressed gene partners. This presents a multi-objective optimization problem because we were trying to maximize two variables, the number of positive partners and the number of negative partners, simultaneously. It is unlikely that a single gene will maximize both of these objectives, so instead of looking for a single solution, we used Pareto optimization, a multi-objective optimization algorithm, to identify the set of genes that most closely maximize both objectives. To illustrate this, we plotted the number of positive partners by the number of negative partners for each gene (Figure 1) and identified the genes that fall along the leading edge of the data, termed the Pareto front. The genes that lie along the Pareto front have more positively and negatively coexpressed gene partners than any gene falling to the left of this curve. We consider each of these genes to be essential and thus a potential drug target.

## Results

### Letrozole induces differential coexpression in ER^+^ breast tumors

Treatment with the aromatase inhibitor letrozole changes gene expression globally, resulting in a marked downregulation of genes involved in cell-cycle processes including mitosis and DNA metabolism and an upregulation of genes involved in wounding and immune responses, skin and vasculature development, and cell adhesion [19].

Building on this knowledge, using transcriptomic profiles for ER^+^ breast tumor biopsy samples collected before (*n* = 58) and after (*n* = 60) a course of neoadjuvant treatment with letrozole, we selected the subset of genes that are differentially expressed for coexpression analysis. These data allowed us to generate two snapshots of gene–gene relationships: those that occurred among these genes in the untreated tumors and those that occurred among these genes in the residual tumors, which have adapted to tolerate the presence of the drug. We defined coexpression as a statistically significant Spearman’s correlation coefficient in a partial correlation model. These specifications allowed us to find both linear and non-linear relationships and to focus on direct gene–gene relationships by excluding gene pairs that are coexpressed due to a common regulator.

**Figure 2:**
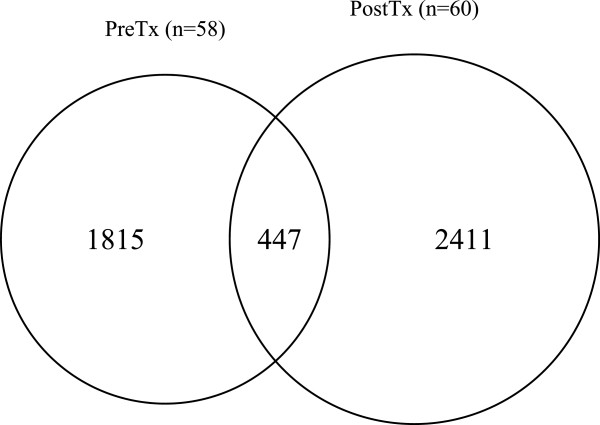
**Differential coexpression among 1,044 genes differentially expressed by letrozole treatment in ER**^**+**^** breast tumor samples.** Coexpression is calculated as the first-order Spearman’s correlation coefficient for each pairwise combination of genes. Approximately 80% of coexpression relationships are found in only one of two treatment conditions and more coexpression relationships are formed among this gene set in the presence of letrozole. PostTx, post-treatment; PreTx, pre-treatment.

We found considerable differential coexpression among genes between the untreated and letrozole-treated tumor samples. Approximately 80% of pairwise relationships occurred in only one of the two treatment conditions (Figure 2). Furthermore, we identified 1.26 times as many pairwise relationships in the letrozole-treated tumor samples as in the untreated tumor samples among the same set of genes. These dynamic coexpression relationships provide evidence of tumor adaptation emphasizing the context-dependent nature of gene–gene relationships and suggesting that the functional relationships among genes change as the tumors adapt to perturbation by the drug.

Each coexpressed gene pair has either a positive connectivity or a negative connectivity based on the sign of the correlation coefficient that connects the two genes. Gene pairs with positive connectivity have expression levels that are directly correlated. Gene pairs with negative connectivity have expression levels that are inversely correlated. In agreement with previous work on coexpression analysis among human genes [35], we identified more coexpression relationships of positive connectivity than negative connectivity in both the untreated and letrozole-treated tumor samples.

Among genes that have sustained coexpression relationships in both the untreated and letrozole-treated tumor samples, the connectivity patterns were conserved, indicating that the nature of the relationships between these genes does not change in the presence of the drug. We removed these common connections leaving only those gene pairs that represent differential coexpression between the two treatment conditions for further analysis.

### Pairwise coexpression relationships are supported by known biological evidence

To confirm that we had identified pairwise relationships with biological relevance we mapped each pair of genes to the Gene Ontology (GO), KEGG, and Reactome databases. We also looked for evidence of functional relationships by querying IMP, a web server that mines empirical data to provide a predictive probability that a pair of genes work together within a biological process. We found that 42% of the coexpressed gene pairs in untreated tumors and 45% of the coexpressed gene pairs in letrozole-treated tumors are supported by at least one of these sources of biological evidence.

Furthermore, we looked for evidence of the biological effects of drug treatment. Among the pathway and process databases, GO has the highest coverage for our gene set (88%) compared to KEGG (38%) and Reactome (35%). So we isolated GO biological process terms that are exclusively represented by gene pairs in the letrozole-treated tumor samples. We found that these processes correspond to both the intended effects and side effects of the drug (Additional file 8). Examples include decreased mitosis, bone density loss [36], hypercholesterolemia [36], arthralgia and myalgia [37,38].

### Adaptive coexpression propounds druggable targets for combination therapy

Gene-wise analysis shows that, regardless of letrozole treatment status, most genes have only a few coexpression partners and a propensity toward relationships of positive connectivity while a few genes have many coexpression partners usually incorporating both positive and negative connectivities (Figure 1). In general, genes tend to form more coexpression relationships in the presence of the drug with a noticeable increase in the number of relationships of negative connectivity.

Our goal was to identify druggable targets that will synergize with neoadjuvant letrozole treatment. Our strategy was to identify the genes that have connectivity patterns consistent with those of essential genes because these are the points at which the tumors are likely to be vulnerable to a targeted attack. Based on empirical data showing a tendency for essential genes to form many relationships of both positive and negative connectivities, termed double connectivity [34,39], we used Pareto optimization (see Methods) to identify essential genes as those that maximize the numbers of positive and negative coexpression relationships, simultaneously.

**Figure 3:**
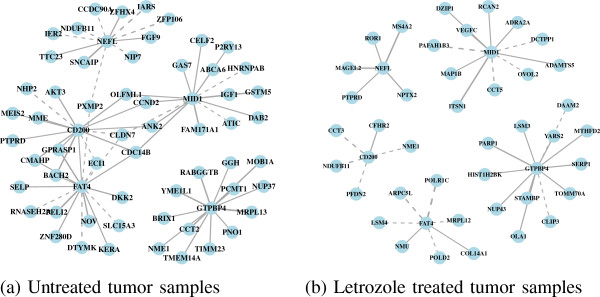
**Coexpression subnetworks for each of the Pareto optimal genes.** **(a)** Genes in untreated tumor samples. **(b)** Genes in letrozole-treated tumor samples. Due to their numbers of positive and negative coexpression partners, GTPBP4, CD200, MID1, FAT4, and NEFL, are likely important for maintaining the tumor phenotype in an estrogen-rich environment. Targeting one or more of these genes concurrently with letrozole treatment may have a synergistic effect resulting in further reductions in tumor volume. Following a 90-day course of letrozole treatment, the number and identity of coexpression partners of these genes changed, illustrating the context-dependent nature of gene–gene associations and suggesting these genes are not as important in an estrogen-depleted environment. Dotted lines indicate negative relationships.

We first identified genes with high double connectivity in the untreated tumors as genes that are likely important for maintaining the tumor phenotype in an estrogen-rich environment. This gene set includes the GTPase GTPBP4, the glycoprotein CD200, the microtubule-associated MID1, the cadherin FAT4, and the neurofilament NEFL (Figure 1a). We see context-dependent associations among these genes and their coexpression partners illustrated by the tendency to form more coexpression relationships prior to letrozole treatment and to associate with a different set of genes under each treatment condition (Figure 3).

Each gene in this set has the potential to be a druggable target. Targeting one or more of these genes concurrently with the inhibition of estrogen signaling, through letrozole treatment, has the potential to enhance letrozole’s ability to reduce tumor volume. There is limited literature regarding the functional role of GTPBP4 in the context of cancer. One report suggests that inhibition of this gene could be effective by showing an inverse relationship between the expression level of GTPBP4 in breast tumors carrying wild-type p53 and patient survival [40].

The other four genes in this set share coexpression relationships to form a connected subnetwork, which suggests that targeting just one of these genes could effectively modulate the expression of the others. Based on their biological roles in the context of cancer, it appears that the inhibition of CD200 and FAT4 would be effective in reducing tumor volume. A series of studies demonstrated that overexpression of CD200 promotes tumor growth and metastasis of breast cancer in immunocompetent mice through suppression of the immune response, a process that can be reversed by treatment with an anti-CD200 monoclonal antibody [41,42]. FAT4 is a member of the Hippo signaling pathway and has been classified as a putative tumor suppressor in breast cancer [43], although, this is a context-dependent designation as it has also been shown to play roles in tumorigenesis and planar cell polarity [44], a process linked to metastasis. FAT4 has been recently implicated as a druggable target [45] but to date there are no drugs that specifically target this gene.

In contrast, the downregulation or loss of MID1 and NEFL has been associated with more aggressive disease. MID1 was recently shown to mediate the ubiquitin-dependent degradation of *α*4 [46], a regulator of mTOR and cell-cycle progression, which is highly expressed in breast cancer [47]. NEFL is an independent prognostic indicator of disease-free survival in early stage breast cancer where low expression correlates with worse outcome [48]. Our coexpression networks show that expression of FAT4 is positively correlated with CD200 and negatively correlated with both MID1 and NEFL suggesting that inhibition of FAT4 may be the optimal target for neoadjuvant co-treatment with letrozole because its modulation may downregulate CD200 while upregulating MID1 and NEFL.

**Figure 4:**
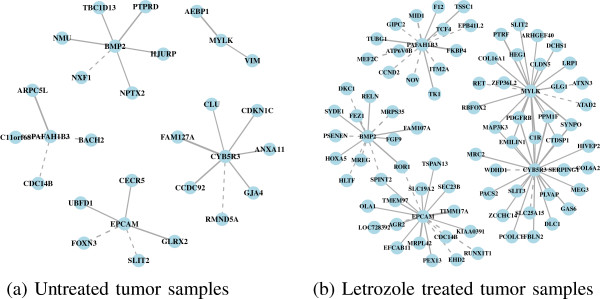
**Coexpression subnetworks for each of the Pareto optimal genes.** **(a)** Coexpression partners of CYB5R3, MYLK, EPCAM, BMP2, and PAFAH1B3, prior to letrozole treatment. **(b)** Following letrozole treatment, the number and identity of coexpression partners of these genes changed, illustrating the context-dependent nature of gene–gene associations and suggesting that these genes may have an important role in maintaining the tumor phenotype in an estrogen-depleted environment. Targeting one or more of these genes sequentially following letrozole treatment, after the tumors have adapted to the drug, may have a synergistic effect resulting in further reductions in tumor volume.

We also identified genes with high double connectivity in the letrozole-treated tumors as genes that are essential in an estrogen-depleted environment. Targeting these genes sequentially after estrogen signaling has been inhibited by letrozole has the potential to reduce further tumor volume by blocking escape pathways as they emerge while the tumors try to adapt to the drug. In the letrozole-treated tumors, the essential gene set includes the enzyme CYB5R3, the kinase MYLK, the antigen EPCAM, the growth factor BMP2, and the acetylhydrolase PAFAH1B3 (Figure 1b). These genes tend to have more coexpression partners following letrozole treatment relative to the untreated tumor samples (Figure 4). And again, the set of genes acting as coexpression partners differs following letrozole treatment, showing the context-dependent nature of gene–gene associations.

The expression levels of CYB5R3 and MYLK are positively correlated. The CYB5R3 gene plays a functional role in redox homeostasis by maintaining the balance of NAD+ /NADH within cells. It has been linked to cancer through its association with mitochondrial dysfunction [49]. Mitochondrial dysfunction promotes tumor growth in a condition-dependent manner [50]. MYLK is associated with breast tumor metastasis through *in vitro* studies showing its role in mediating migration and invasion of the MDA-MB-231 cell-line [51] and the intravasation of breast cancer cells through an endothelial cell layer [52]. Inhibition of either of CYB5R3 or MYLK could be effective at halting tumor progression because the inhibition of one of these genes should modulate the expression of the other.

The expression levels of EPCAM and BMP2 are negatively correlated. EPCAM is a cell-adhesion molecule that has been associated with cell signaling, proliferation, differentiation, migration and metastasis [53] and used as a marker of both primary tumors and circulating tumor cells for patients with breast cancers and other endothelium-derived tumors [54,55]. Silencing EPCAM gene expression in breast tumor cell lines *in vitro* results in a dramatic decrease in metabolic activity, cell migration and invasion [56]. BMP2, a member of the TGF *β* superfamily, is a target gene of ER signaling, which is downregulated in the presence of estrogen [57]. By forming a heterodimer with BMP7, BMP2 acts as a TGF *β* antagonist and prevents bone metastases in a mouse model of breast cancer [58]. Due to the negative coexpression relationship that exists between EPCAM and BMP2, inhibition of EPCAM may upregulate BMP2 to contribute to the prevention of metastasis. Notably, several drugs have been developed to target EPCAM as cancer therapeutics [59,60]. To our knowledge, the association between PAFAH1B3 and breast cancer is novel.

### Replication highlights biologically relevant and novel coexpression relationships

**Figure 5:**
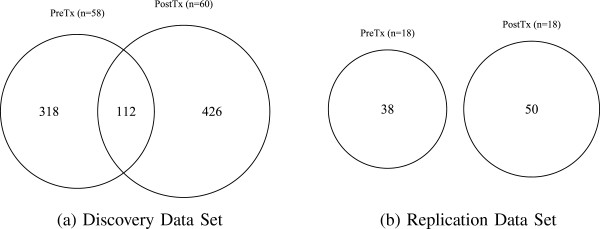
**Differential coexpression among 263 genes differentially expressed by letrozole treatment.** Two independent data sets of ER^+^ breast tumor samples were used. Coexpression was calculated as the first-order Spearman’s correlation coefficient for each pairwise combination of genes. In both the **(a)** discovery and **(b)** validation data sets, most coexpression relationships are unique to one of the two treatment conditions with an increase in the number of letrozole-induced coexpressed gene pairs. PostTx, post-treatment; PreTx, pre-treatment.

To determine if coexpression relationships induced by letrozole treatment are generalizable, we did a replication analysis with an independent data set. This data includes transcriptomic profiles for 18 ER^+^ breast tumor biopsy samples collected before and after a course of neoadjuvant treatment with letrozole. For consistency, we used only the subset of genes that were differentially expressed by letrozole treatment in both data sets resulting in a set of 263 genes for coexpression analysis. Confirming our earlier finding, there was patent differential coexpression among this set of genes for both data sets, with an increase in the number of pairwise relationships among genes in the letrozole-treated samples (Figure 5). With fewer samples in the replication data, we had limited statistical power to detect patterns of coexpression; however, those relationships that do replicate provide validation for letrozole-induced tumor adaptation.

We validated six gene–gene relationships induced by letrozole treatment (Table 1). One gene pair is supported by strong biological evidence and the other five gene pairs validate novel relationships. To attach functional meaning to these novel findings we generated functional subnetworks in IMP that incorporate additional genes to make connections between the coexpressed gene pairs. The first validated relationship is a positive connection between the ribonucleotide reductase RRM2 and the DNA topoisomerase TOP2A, two genes that map to the DNA replication pathway. They have a high probability of a functional interaction in IMP (0.88) and are downregulated by letrozole treatment in agreement with the effects of blocking ER signaling [61].

**Table 1:** Replication of letrozole-induced coexpression relationships

**Gene 1**	**Gene 2**	**Gene 1 expression change**	**Gene 2 expression change**	**PCOR coefficient discovery data**	**PCOR coefficient replication data**	**GO term**	**KEGG term**	**REACTOME term**	**IMP**
RRM2	TOP2A	*↓*	*↓*	0.44	0.90	DNA replication	-	-	0.88
LINC00341	RUNX1T1	*↑*	*↑*	0.34	0.78	-	-	-	-
MN1	SPARC	*↑*	*↑*	0.45	0.76	-	-	-	-
FBLN1	FLRT2	*↑*	*↑*	0.43	0.70	-	-	-	-
MEF2C	LINC00341	*↑*	*↑*	0.37	0.66	-	-	-	-
FSTL1	PDGFRL	*↑*	*↑*	0.47	0.63	-	-	-	0.10

We identified coexpression relationships among a set of 263 genes that were differentially expressed in two independent data sets of breast tumor biopsies following 90 days of letrozole treatment. The coexpressed gene pairs that validated are shown here with their pathway annotations and IMP score, which can be interpreted as the predictive probability that the two genes have a functional interaction based on a large compendium of empirical data. GO, Gene Ontology; IMP, Integrated Multi-species Prediction; KEGG, Kyoto encyclopedia of genes and genomes; PCOR, Partial correlation coefficient.

**Figure 6:**
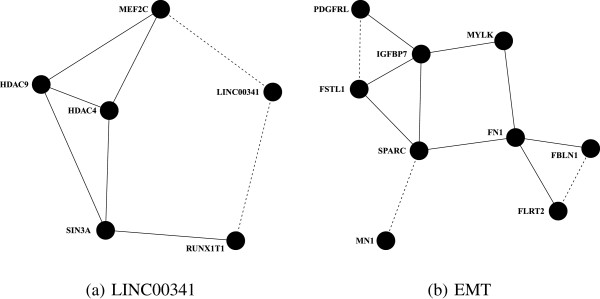
**Letrozole-induced tumor adaptation is validated through replication of coexpressed gene pairs in the residual tumors.** To make functional connections among gene pairs that have not been annotated to the same biological pathways, we used IMP, a web-based tool that mines empirical data to provide a predictive probability that two genes have a functional relationship. **(a)** We identified a functional subnetwork that implicates LINC00341, a long-non-coding RNA of unknown function, in ER-mediated repression. **(b)** We uncovered a functional subnetwork of genes associated with the EMT, a process that promotes tumor metastasis. Dotted lines indicate coexpression relationships. Solid lines indicate functional relationships determined by IMP with a predictive probability of at least 0.70. EMT, epithelial-to-mesenchymal transition.

The next two validated gene pairs involve a long non-coding RNA, LINC00341, of unknown function. LINC00341 is coexpressed with RUNX1T1, a proto-oncogene and transcriptional repressor, which interacts with DNA-bound transcription factors, and MEF2C, a transcription factor involved in myogenesis and muscle cell differentiation maintenance. Functionally, RUNX1T1 and MEF2C are linked through two intermediates, SIN3A and both HDAC4 and HDAC9, all of which are transcriptional repressors (Figure 6a). This suggests that LINC00341 is part of a complex that regulates transcription of MEF2C, a gene that has previously been shown to be regulated by long non-coding RNAs [62].

The remaining three validated gene pairs constitute a subnetwork representative of the epithelial-to-mesenchymal transition (EMT), a process associated with wound healing and metastasis (Figure 6b). The two key genes that complete the functional subnetwork among these gene pairs, the glycoproteins FN1 and SPARC, are bona fide markers of EMT [63,64]. FN1 functionally connects a pair of coexpressed glycoproteins, FBLN1 and FSTL1, and SPARC is connected to IGFBP7, a tumor suppressor, which creates a functional link between a candidate tumor suppressor, PDGFRL, and a mesenchymal factor, FSTL1. In addition to FN1 and SPARC, other well-established EMT-associated genes are also upregulated in these tumor samples following letrozole treatment including TWIST1, SNAI2, ZEB1, and ZEB2 [65]. We did not identify any of these replicated coexpression relationships in the untreated tumor samples as evidence that the residual tumor cells have undergone a functional reorganization during adaptation to tolerate the presence of the drug.

## Discussion

Here, we introduced a method to prioritize genes that have coexpression and connectivity patterns consistent with those of essential genes [34] as potential drug targets in the design of rational combination therapies for the treatment of cancer. We applied this method to predict combination therapy targets based on the adaptive response of ER^+^ breast tumors to neoadjuvant treatment with the aromatase inhibitor letrozole. We used coexpression as a proxy for functional relationships and found that adaptation to drug perturbation is evident in the differential coexpression patterns we observed between the untreated and letrozole-treated tumor samples. This is consistent with previous work showing that functional relationships among genes are dependent on the cellular state and local environment and reflected in patterns of coexpression [10].

We confirmed that many of the coexpressed gene pairs we identified have known biological relevance, but we also found pairs that are not yet annotated to the same processes or pathways and do not yet have empirical evidence that predicts a functional relationship. Perhaps the most obvious reason for this is annotation bias, which occurs because well-studied genes are assigned many annotations while the understudied genes may not be annotated at all [66,67]. In our analysis, 26% of the genes have one or fewer annotations. Presumably, many of these genes are multifunctional, serving to connect related biological pathways that will not be revealed through annotation analysis alone. This is one of the reasons we incorporated IMP as a discovery tool, to move beyond curated annotations to find functional relationships supported by empirical data.

Repeated sampling of tumors before and after letrozole treatment allowed us to capture dynamic changes in gene expression and coexpression, illustrating changes in the functional relationships among genes that are induced by the drug. In this way, the adaptive response becomes a process that can be exploited to identify context-dependent targets. In total, we have identified ten Pareto optimal genes as potential targets for use in combination with letrozole. Of these genes, EPCAM stands out because opportunely, several monoclonal antibodies have already been developed against EPCAM as cancer therapeutics, including the well-tolerated, fully humanized version, adecatumumab [59]. Inhibition of EPCAM with adecatumumab has only been tested in patients with advanced disease. As a single agent, adecatumumab shows activity in metastatic breast cancer, but does not lead to tumor regression [68]. The combination of docetaxel and adecatumumab in a Phase IB trial achieved a clinical benefit, defined as a complete or partial response or stable disease, in 44% of patients with relapsed or refractory advanced-stage breast cancer [69].

Based on our findings, the addition of adecatumumab following an initial period of letrozole treatment should enhance the anti-tumor effects of letrozole alone. Suitably, recent trials have demonstrated that patients continue to derive a clinical benefit from neoadjuvant letrozole for up to one year of treatment [70-73], making sequential therapy a fitting option. Moreover, metastasis is virtually prevented in mice when treated with a murine-specific version of adecatumumab [74], which suggests that this combination has the potential to be a long-term treatment strategy for the management of ER^+^ breast cancer as a chronic condition in elderly patients [75].

Despite differences in inclusion criteria and the limited sample size of the replication data, we were able to replicate six letrozole-induced coexpression relationships as validation of letrozole-induced adaptation. Two of the novel relationships that replicate provide clues about the function of the uncharacterized long non-coding RNA LINC00341. We have shown that LINC00341 is coexpressed with both RUNX1T1 and MEF2C (Figure 6a). RUNX1T1 is part of a corepressor complex that interacts with SIN3A *in vivo*[76]. SIN3A interacts with HDACs 4 and 9, specifically binding the catalytic domain of HDAC 9 in cells derived from B-cell tumors [77]. HDAC4 and HDAC9 also physically interact with MEF2C repressing MEF2C-dependent transcription [78,79]. Inhibition of SIN3 activity in breast cancer cells leads to the derepression of silenced genes, such as ESR1 *α*, restoring sensitivity to tamoxifen treatment [80]. Through the same mechanism, inhibiting HDACs in combination with letrozole is more effective at suppressing tumor growth in a xenograft model than either treatment alone [81]. In this context, through guilt-by-association [11], it appears that LINC00341 may play a role in ER-mediated transcriptional repression.

We also showed that three validated gene pairs constitute a subnetwork representative of the EMT (Figure 6b). This is in agreement with a previous study showing that breast tumors contain cells with both epithelial and mesenchymal markers, the latter being associated with residual tumor following either chemotherapy or letrozole treatment in breast cancer [20]. EMT-derived cells can differentiate into mature osteoblasts, adipocytes or chondrocytes, and they have the ability to invade and migrate, homing toward wound sites [82] and participating in the invasion-metastasis cascade [83]. The SPARC and FN1 genes have an established association with EMT [63,64]. IGFBP7, a secreted tumor suppressor, can discriminate circulating endothelial cells of cancer patients from those of healthy donors [84] and, in this context, it functionally connects SPARC, PDGFRL, a gene that is highly expressed as primary melanomas transition into metastatic melanomas [85], FSTL1, a diffusible mesenchymal factor that can independently determine the cell fate of the endothelium [86], and MYLK, a multifunctional kinase that is involved in epithelial cell survival, is required for epithelial wound healing, and is included in our list of Pareto optimal genes for the letrozole-treated tumors.

Notably, although EPCAM was not in the subset of 263 genes, it is also a marker of EMT and circulating endothelial cells [54,87]. Suppression of EPCAM attenuates tumor progression and downregulates transcription factors that are involved in EMT reprogramming [88]. We have validated the EMT pathway as a biological process involved in tumor adaptation to letrozole treatment and identified two potential targets within this pathway, MYLK and EPCAM, in the discovery data set as letrozole-induced essential genes, whose targeting should have a synergistic effect with neoadjuvant letrozole treatment.

We have focused on using the adaptive process at a single treatment time point to identify a letrozole-induced essential gene as a second target for sequential therapy. Because tumors comprise heterogeneous cell populations, it is likely that letrozole acts as a selective pressure, changing the proportions of clonal populations within the tumor, in addition to modulating gene expression within individual cells. This combination of tumor evolution and adaptation provides the tumor with a plethora of ways to resist the effects of the drug. In light of this, we believe this approach will reach its full potential when applied serially throughout the course of treatment with the sequential addition of drugs until the tumor has regressed enough to be completely resected or until there is no evidence of disease. If we can understand how relationships between genes change in response to a given treatment, we can plan interventions that will interfere with the adaptation process, preventing the development of resistance.

## Conclusions

The advantage of molecularly targeted drugs is that they selectively act on cancerous cells leading to fewer side effects and better patient outcomes. However, tumors are dynamic living systems that modulate gene expression and coexpression relationships as part of an adaptive response that facilitates robustness in the face of these targeted perturbations. By focusing on patterns of coexpression in breast tumors, before and after letrozole treatment, we were able to capture this adaptive response and identify tumor-essential genes and letrozole-induced tumor-essential genes as potential candidates for combination therapy with neoadjuvant letrozole treatment. Given complete data sets of serially sampled tumors throughout a course of treatment, this approach could be an effective means of designing adaptive treatment strategies that respect the context-dependent functions of genes and the resilience of tumor cells, providing an opportunity to refine further the process of personalized medicine by pairing targeted drugs with evolving tumor phenotypes.

## Abbreviations

EMT: epithelial-to-mesenchymal transition; ER: estrogen receptor; FDR: false discovery rate; Gene Expression Omnibus; GEO: GO, Gene Ontology; IMP: Integrated Multi-species Prediction; KEGG: Kyoto encyclopedia of genes and genomes; limma: linear models for microarray data.

## Competing interests

The authors declare that there are no competing interests.

## Authors’ contributions

NP conceived the study and performed the analyses. NP, CG and JM designed the study and drafted the manuscript. All authors read and approved the final manuscript.

## Supplementary Material

Additional file 1 Permutation testing for coexpression. Click here for file

Additional file 2 Coexpressed gene pairs in the discovery data set: untreated. Click here for file

Additional file 3 Coexpressed gene pairs in the discovery data set: letrozole treated. Click here for file

Additional file 4 Coexpressed gene pairs in the discovery data set (replication study): untreated. Click here for file

Additional file 5 Coexpressed gene pairs in the discovery data set (replication study): letrozole treated. Click here for file

Additional file 6 Coexpressed gene pairs in the replication data set: untreated. Click here for file

Additional file 7 Coexpressed gene pairs in the replication data set: letrozole treated. Click here for file

Additional file 8 Table of GO terms that are exclusively shared by coexpressed gene pairs in the letrozole-treated tumors and their associated biological effects. Click here for file
