# Supplementary material for: Predicting targeted drug combinations based on Pareto optimal patterns of coexpression network connectivity
Source: Genome Med. 2014 Apr 30;6(4):33. doi: 10.1186/gm550 (PMC4062052; doi:10.1186/gm550)
Supplement: Additional file 1 — Permutation testing for coexpression. [file gm550-S1.pdf]

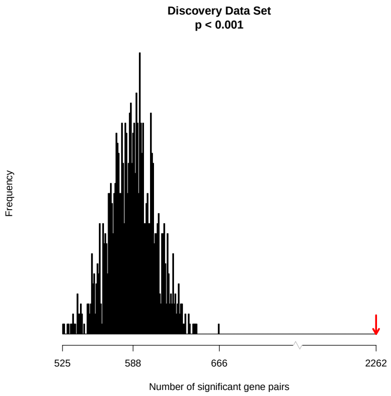

(a) Pre-Tx (1044 genes)

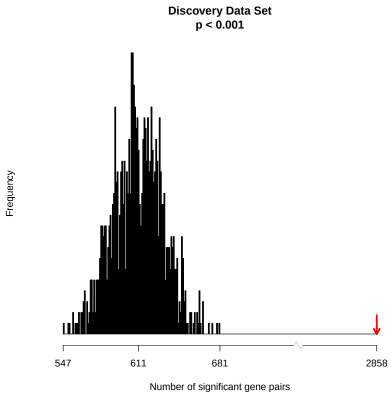

(b) Post-Tx (1044 genes)

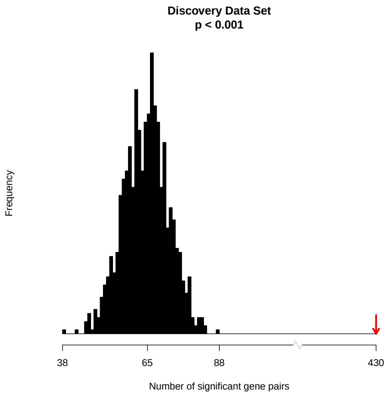

(c) Pre-Tx (263 genes)

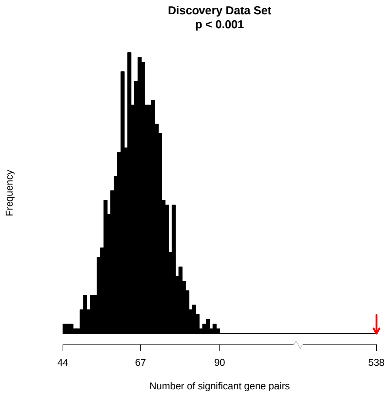

(d) Post-Tx (263 genes)

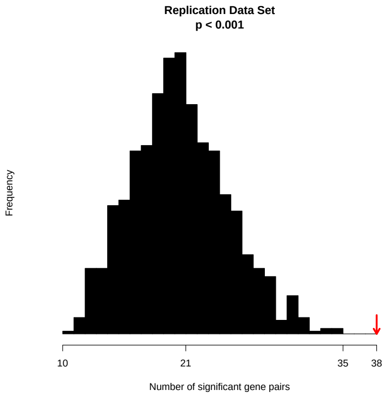

(e) Pre-Tx (263 genes)

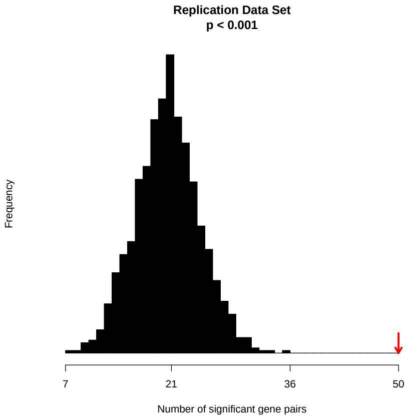

(f) Post-Tx (263 genes)

Additional file 1 — **Permutation testing for coexpression.** Permutation tests were designed to randomize the expression values for each gene, across samples, within each time point. After randomization, we calculated coexpression by the 1st-order Spearman's correlation coefficient. We counted the number of significant results ($\alpha = 0.001$) then repeated 1000 times to generate a null distribution. Arrows indicate the number of observed coexpression relationships for a given data set.
