# Supplementary material for: Predicting targeted drug combinations based on Pareto optimal patterns of coexpression network connectivity
Source: Genome Med. 2014 Apr 30;6(4):33. doi: 10.1186/gm550 (PMC4062052; doi:10.1186/gm550)
Supplement: Additional file 8 — Table of GO terms that are exclusively shared by coexpressed gene pairs in the letrozole-treated tumors and their associated biological effects. [file gm550-S8.pdf]

Additional file 8 - Table of GO terms that are exclusively shared by coexpressed gene pairs in the letrozole-treated tumors and their associated biological effects.

Gene Symbol		Log Fold-change		pcor	GO Term	Effect
Gene 1	Gene 2	Gene 1	Gene 2			
CYB5R3	SERPING1	0.34	0.64	0.33	blood circulation	
A2M	VWF	0.66	0.57	0.71	blood coagulation, intrinsic pathway	Blood clotting
TGFBR2	CAV1	0.34	0.59	0.57	vasculogenesis	
COL1A1	MMP2	0.79	0.94	0.53	bone trabecula formation	
					face morphogenesis	
					intramembranous ossification	
FGF9	BMP2	0.17	0.12	0.38	cell-cell signaling	Bone density
					osteoblast differentiation	
					positive regulation of gene expression	
COL1A1	MN1	0.79	0.28	0.34	intramembranous ossification	
INHBB	TCF7L2	0.47	0.40	0.33	fat cell differentiation	Cholesterol homeostasis
ACAT2	AGPS	-0.27	-0.20	0.42	lipid metabolic process	
ADRA2A	TCF7L2	0.33	0.40	0.42	glucose homeostasis	Glucose homeostasis
GPR116	LPHN2	0.28	0.14	0.35	neuropeptide signaling pathway	
MEOX2	CAV1	0.26	0.59	0.59	skeletal muscle tissue development	
KCNMB4	NPTX2	0.30	0.18	0.52	synaptic transmission	
NEFL	NPTX2	0.09	0.18	0.40	synaptic transmission	
FOS	JUN	1.10	0.48	0.44	cellular response to calcium ion	
					MyD88-dependent toll-like receptor signaling pathway	
					MyD88-independent toll-like receptor signaling pathway	
					regulation of sequence-specific DNA binding transcription factor activity	Arthalgia and myalgia
					response to lipopolysaccharide	
					response to mechanical stimulus	
					response to organic cyclic compound	
					SMAD protein signal transduction	
					stress-activated MAPK cascade	
					toll-like receptor 1 signaling pathway	
					toll-like receptor 2 signaling pathway	
					toll-like receptor 3 signaling pathway	
					toll-like receptor 4 signaling pathway	
					toll-like receptor signaling pathway	
					Toll signaling pathway	
FYN	HLA-DMA	0.25	0.42	0.36	T cell costimulation	
					T cell receptor signaling pathway	
UBE2C	CDC20	-0.60	-0.68	0.43	activation of anaphase-promoting complex activity	
					mitotic cell cycle spindle assembly checkpoint	
					negative regulation of ubiquitin-protein ligase activity involved in mitotic cell cycle	
					positive regulation of ubiquitin-protein ligase activity involved in mitotic cell cycle	
CHEK1	BIRC5	-0.08	-0.20	0.47	G2/M transition of mitotic cell cycle	
CCNB2	CDK1	-0.59	-0.38	0.47	G2/M transition of mitotic cell cycle	
MELK	CDK1	-0.72	-0.38	0.40	G2/M transition of mitotic cell cycle	
ORC6	GINS1	-0.16	-0.67	0.34	S phase of mitotic cell cycle	
NCAPH	NUSAP1	-0.17	-0.55	0.47	mitotic chromosome condensation	Cell cycle
KIFC1	NEK2	-0.15	-0.10	0.46	mitotic sister chromatid segregation	
MCM6	POLE2	-0.29	-0.24	0.36	DNA-dependent DNA replication initiation	
					M/G1 transition of mitotic cell cycle	
					S phase of mitotic cell cycle	
MCM4	GINS2	-0.15	-0.59	0.38	DNA strand elongation involved in DNA replication	
					S phase of mitotic cell cycle	
FOXM1	TRIP13	-0.22	-0.43	0.48	transcription from RNA polymerase II promoter	
MEIS2	NFIB	0.68	0.55	0.40	transcription from RNA polymerase II promoter	
EGR1	FOS	0.69	1.10	0.36	transcription from RNA polymerase II promoter	
NR3C1	MEF2C	0.29	0.26	0.35	transcription from RNA polymerase II promoter	
POLR2H	POLR1C	-0.32	-0.21	0.38	termination of RNA polymerase I transcription	
					transcription elongation from RNA polymerase I promoter	
					transcription from RNA polymerase I promoter	
					transcription initiation from RNA polymerase I promoter	
MECOM	FOXO1	0.29	0.40	0.39	regulation of cell proliferation	
TXNIP	TGFBR2	0.50	0.34	0.38	regulation of cell proliferation	
LSM3	DCP1A	-0.22	0.20	-0.34	exonucleolytic nuclear-transcribed mRNA catabolic process involved in deadenylation-dependent decay	
					nuclear-transcribed mRNA catabolic process, deadenylation-dependent decay	
FRZB	HTR2B	0.38	0.22	0.40	neural crest cell differentiation	
FRZB	MEF2C	0.38	0.26	0.36	neural crest cell differentiation	
S1PR1	MEF2C	0.41	0.26	0.47	neuron differentiation	
TCF4	ZEB1	0.39	0.18	0.48	positive regulation of neuron differentiation	Epithelial mesenchymal transition
COL1A1	COL1A2	0.79	0.61	0.50	skin morphogenesis	
NXN	TGFB1I1	0.35	0.46	0.57	Wnt receptor signaling pathway	
LOX	SPARC	0.17	0.57	0.34	lung development	
NR3C1	PER3	0.29	0.28	0.44	circadian rhythm	
GAS6	PDGFRB	0.40	0.44	0.39	positive regulation of ERK1 and ERK2 cascade	
ANXA1	CXCL12	0.57	0.75	0.43	negative regulation of apoptosis	
FOXO1	TCF7L2	0.40	0.40	0.37	anti-apoptosis	
CETN2	GTF2H5	-0.36	-0.35	0.36	nucleotide-excision repair	
FAT1	SEMA5A	0.41	0.35	0.42	cell-cell signaling	
GEM	ANXA1	0.90	0.57	0.36	cell surface receptor linked signaling pathway	
CRYAB	CAV1	0.28	0.59	0.40	protein homooligomerization	Other
DNM1	EHD2	0.59	0.29	0.41	endocytosis	
JAM3	MTL5	0.45	-0.50	-0.36	spermatogenesis	
KLF2	JUNB	0.38	0.41	0.37	in utero embryonic development	
GAS1	CCND2	0.24	0.11	0.36	positive regulation of epithelial cell proliferation	
TXNIP	DUSP1	0.50	0.45	0.35	response to calcium ion	
					response to estradiol stimulus	
					response to hydrogen peroxide	
					response to oxidative stress	
DUSP1	JUN	0.45	0.48	0.43	response to hydrogen peroxide	
TXNIP	CCNB1	0.50	-0.80	-0.34	response to mechanical stimulus	
